# METTL16 regulates the mRNA stability of FBXO5 via m6A modification to facilitate the malignant behavior of breast cancer

**DOI:** 10.1186/s40170-024-00351-5

**Published:** 2024-07-25

**Authors:** Runying Wang, Xingjie Gao, Luhan Xie, Jiaqi Lin, Yanying Ren

**Affiliations:** 1 MOE Key Laboratory of Bio-Intelligent Manufacturing, School of Bioengineering, Dalian University of Technology, Dalian City, 116024 Liaoning Province P.R. China; 2 Department of Biotechnology, College of Basic Medical Sciences, Dalian Medical University, No.9 West Section, Lvshun Road, Dalian City, 116044 Liaoning Province P.R. China; 3 Deparment of Pathology and Forensic Medicine, Dalian Medical University, No.9 West Section, Lvshun Road, Dalian City, 116044 Liaoning Province P.R. China; 4 Hernia and Colorectal Surgery, The Second Hospital of Dalian Medical University, Dalian City, 116023 Liaoning Province P.R. China

**Keywords:** Breast cancer, m6A modification, METTL16, FBXO5

## Abstract

**Background:**

N6-methyladenosine (m6A) regulates the progression of breast cancer (BC). We aimed to investigate the action and mechanism involved of methyltransferase-like protein 16 (METTL16) in BC growth and metastasis.

**Methods:**

RT-qPCR, immunoblotting, and IHC were performed to test the levels of gene expression. CCK-8, clone formation, wound healing, and transwell assays were applied to measure the cell proliferation, migration, and invasion. m6A RNA methylation and MeRIP assay were utilized to confirm the m6A level of total RNA and FBXO5 mRNA. RIP was utilized to ascertain the interaction between METTL16 and FBXO5 mRNA. The in vivo murine subcutaneous tumor and metastasis model were constructed to further confirm the action of METTL16.

**Results:**

METTL16 was overexpression in BC cells and tissues. Inhibition of METTL16 restrained the growth and metastasis of BC. Furthermore, the METTL16 level and FBXO5 level was positively correlated in BC tissues, and METTL16 aggrandized the stability of FBXO5 mRNA depending on the m6A modification. Overexpression of FBXO5 antagonized the restrained function of METTL16 knockdown on BC cells’ proliferation, migration, invasion, and EMT.

**Conclusion:**

METTL16 boosts the mRNA stability of FBXO5 via m6A modification to facilitate the malignant action of BC in vitro and in vivo, offering new latent targets for cure of BC.

**Supplementary Information:**

The online version contains supplementary material available at 10.1186/s40170-024-00351-5.

## Introduction

Breast cancer (BC) is the most usual malignant tumor and the fifth leading reason of cancer-related death in the world [1]. BC is a group of molecularly heterogeneous diseases, and its treatment options depend on various factors, such as the disease type, disease stage, and human epidermal growth factor receptor 2 (HER2)’s level [2]. The combination of surgery, radiotherapy, chemotherapy, endocrine and targeted therapy is commonly used for the treatment of BC [2, 3]. These treatment approaches have enhanced the livability of BC patients, while some patients still experience the risk of recurrence and death due to the occurrence of metastasis [2, 4]. Hence, it is still necessary to probe the mechanism of BC development to seek new latent diagnosis and treatment methods.

N6-methyladenosine (m6A) involved in many RNA processing progresses, such as splicing, transport, translation and degradation [5]. Xu et al. found that LATS1, which is positively regulated by m6A modification, performs a facilitating function in the proliferation and glycolysis metabolism of BC [6]. Additionally, the inhibition of m6A modification of PD-L1 can motivate tumor immune surveillance in BC [7]. This indicated that m6A modification performs a vital function in the occurrence and development of BC. Methyltransferase-like protein 16 (METTL16), an RNA methyltransferase that can mediate the m6A modification of RNA, has been reported to regulate tumorigenesis [8]. Research revealed that METTL16 can promote the progression of osteosarcoma by degrading VPS33B mRNA [9]. METTL16 can also augment the metabolic reprogramming of colorectal cancer via enhancing the mRNA stability of SOGA1 [10]. Moreover, reported showed that METTL16 level is enhanced in BC tissues and its inhibition lessened the tumor growth of BC in vivo [11]. While the downstream targets of METTL16 are still unclear.

F-Box Protein 5 (FBXO5, also named Emi1) is a regulator of anaphase promoting complex/cyclosome (APC/C) activity in mitotic and meiotic cell cycle regulation and was reported to be overexpressed in a variety of cancer cells and enhance cancer progress [12–14]. For example, inhibition of FBXO5 restrained the proliferation of lung cancer cells [15]. FBXO5 knockdown can inhibit glioma proliferation, migration and invasion [16]. Some research also found that FBXO5 level was increased in BC tissues and relevant to the disease stage and poor prognosis [17, 18], but the function of FBXO5 in BC is currently unknown.

In this study, we proved that METTL16 increases the mRNA stability of FBXO5 through m6A modification and promotes the malignant behavior of BC, revealing the role and regulatory mechanism of METTL16 in BC and providing new potential target for the diagnosis and cure of BC.

## Methods

### Tissues collection

The BC and adjacent tissues (*n* = 20) were gained from the BC patients who have not undergone any treatment in Dalian University of Technology from August 2022 to February 2023. All people in the research participated voluntarily and signed the informed consent. All experimental schemes were agreed via the Ethics Committee of Dalian University of Technology.

### Cell culture transfection and infection

BC cells including MDA-MB-231 and MDA-MB-453 were cultivated in DMEM (Gibco) appended with 10% fetal bovine serum (FBS, Gibco), penicillin/streptomycin (Gibco), and humidity of 95% air and 5% CO_2_ at 37 °C. For the overexpression of FBXO5, FBXO5 overexpression vectors (pc-FBXO5) and the matching negative vectors (pc-NC) were established via GenePharma (Shanghai, China). The Lipofectamine 3000 (Invitrogen, USA) was applied to transfect these vectors into BC cells. For the METTL16 knockdown, METTL16 knockdown lentivirus (LV-sh-METTL16) and the matching negative lentivirus (LV-sh-NC) were established via GenePharma. The lentivirus was infected in BC cells and the infected cells were screened in DMEM with puromycin (3 µg/mL, Sigma-Aldrich) for 14 d to construct the stably cell of METTL16 knockdown.

### Reverse transcription-quantitative polymerase chain reaction (RT-qPCR)

Using Trizol (Invitrogen) to separate the overall RNA from the above treated BC cells. The SuperScript™ VILO™ cDNA Synthesis Kit (Thermo Fisher Scientific, USA) was applied to reversed overall RNA into cDNA according to the instructions. Then, the mRNA level of METTL16 and FBXO5 were probed via qPCR with SYBR Green PCR Master Mix (Takara, China) on the 7900HT Fast Real-Time PCR System (Applied Biosystems, USA). The reference gene for normalization was GADPH. The shift of the mRNA level of METTL16 and FBXO5 were quantified via 2^−∆∆Ct^ method. The Forward (**F**) and Reverse (R) primer sequences of METTL16, FBXO5, and GAPDH were offered (5’-3’): METTL16-F: CTCTGACGTGTACTCTCCTAAGG,

METTL16-R: TACCAGCCATTCAAGGTTGCT;

FBXO5-F: GCTGTCATGTATTGGGTCACC,

FBXO5-R: GTCTACTGGTCTCTAGTGCTTCT;

Precursor FBXO5-F: TGCCAGTTGTGTGTATGTGT,

Precursor FBXO5-R: CCTGCACGTACTTAAACAAAGCA;

GAPDH-F: GGAGCGAGATCCCTCCAAAAT,

GAPDH-R: GGCTGTTGTCATACTTCTCATGG.

### Immunoblotting

The overall protein from the above treated cells and tissues were isolated using RIPA lysate (Beyotime, China) and the density of overall protein was probed via BCA kit (Beyotime). Overall protein (25 µg) was isolated via SDS-PAGE and electrotransferred onto PVDF membrane (Millipore, USA) at 4 °C. Afterwards, 5% skim milk was applied to incubate the PVDF membrane placed in at 25 °C for 1 h. The primary antibodies were then cultivated with the PVDF membrane all-night at 4 °C. The membrane was placed in specific secondary antibody for a further 2 h at 25 °C. Finally, using the enhanced chemiluminescence (ECL) system (Thermo Fisher Scientific) to tested the protein bands and using Image J to analyse the bands. The above antibodies including anti-METTL16 (ab252420, 1:1000), anti-FBXO5 (ab187144, 1:5000), anti-E-cadherin (E-cad, ab231303, 1:1000), anti-N-cadherin (N-cad, ab76011, 1:5000), anti-Vimentin (ab92547, 1:2000), anti-GAPDH (ab9485, 1:2000), and secondary antibody (ab6721, 1:10000) were acquired from Abcam (USA).

### Cell counting kit-8 (CCK-8) assay

A 96-well plate was applied to seed BC cells from each treatment group (at a denseness of 7.5 × 10^3^ cells) and cultivated for 24 h, 48 h, and 72 h. Following this, the supernatant was dislodged, and 10 µL of CCK-8 was appended before being placed for 2.5 h. Then, using SpectraMax 190 microplate reader (Molecular Devices, LLC., USA) to probe the absorbance at 450 nm.

### Clone formation assay

A 6-well plate was applied to seed BC cells from each treatment group (at a denseness of 500 cells) and cultivated for 14 d. Using 4% polyformaldehyde to fix the cells for 15 min and using 1% crystal violet (Sigma-Aldrich) to stain the cells for 10 min. Then, the cells were rinsed with running water slowly and visualized under an AX-70 fluorescent microscope (Leica Microsystems Inc., German).

### Wound healing assay

BC cells from each treatment group (at a denseness of 2 × 10^5^ cells) were planted in 12-well plates and cultivated until confluent. Monolayers were scratched using a gun tip of 200 µL, washed by PBS, and cultivated in serum-free DMEM for 1 d. The width of scratches was observed and probed by an AX-70 fluorescent microscopy at 0 h and 24 h, respectively.

### Transwell assay

First, adding the Matrigel (BD Biosciences) to the Transwell upper chamber. 4 h later, BC cells (2.5 × 10^4^ cells) of each group were planted into the upper chamber and cultivated in serum-free DMEM. Then, the lower chamber was appended DMEM possessing 10% FBS. After 24 h cultivation, using methanol to fixed the cells in the lower side of the upper chamber for 0.5 h, and then using the 0.1% crystal violet (Thermo Fisher Scientific) to dye the cells for 1 h. Finally, the invasive cells were monitored under an AX-70 fluorescent microscopy.

### m6A RNA methylation assay

Overall m6A level of RNA was tested by m6A RNA Methylation Assay kit (Abcam). Concretely, the Trizol (Invitrogen, USA) was applied to separate the overall RNA from BC cells and tissues. Overall RNA (200 ng) was appended in the well including Binding Solution and maintained at 37 °C for 1.5 h. After cleaned with Wash Buffer 3 times, the Diluted Capture Antibody was added the wells and placed at 25 °C for 60 min. Afterwards, the wells were cleaned with Wash Buffer and append Diluted Detection Antibody at 25 °C for 30 min. The wells were cleaned using Wash Buffer and added Diluted Enhancer Solution at 25 °C for 0.5 h. The Developer Solution was added in the wells to maintain 5 min and the Stop Solution was added to stop the reaction. The absorbance at 450 nm was tested using the SpectraMax 190 microplate reader.

### RNA immunoprecipitation (RIP) and methylated RIP (MeRIP) assay

RIP assay kit (Millipore, USA) was applied to assess the FBXO5 mRNA’s m6A level and the binding of METTL16 and FBXO5 mRNA. BC cells were washed twice in PBS and lysed in Lysis Buffer. Then, the supernatant was harvested after 14,000×g centrifugation for 10 min. Meanwhile, the Protein A/G agarose beads were mixed with anti-m6A (ab208577, Abcam), anti-METTL16 (ab252420, Abcam), or anti-IgG (ab172730, Abcam) antibodies for 1 h and the complex of Protein A/G agarose beads and antibodies was harvested after 4000×g centrifugation for 1 min. The supernatants were added to the complex of Protein A/G agarose beads and antibodies and placed for 12 h at 4 °C. Using the Trizol (Invitrogen) to isolate the immune-precipitate mRNA and using RT-qPCR to test the FBXO5 mRNA’s level.

### RNA stability assay

To test the impact of METTL16 on the FBXO5 mRNA’ stability, the BC cells were treated in actinomycin D (2 µg/mL) after knockdown of METTL16. Then, using RT-qPCR assessed the FBXO5 mRNA level after 0, 2, 4, 6, and 8 h.

### Animal experiment

The female BALB/c nude mice (5–6 weeks) were gained from Hunan Slyke Jingda Experimental Animal Co., LTD (China) and maintained in cages with free access to food and water. The lentivirus carrying sh-NC (LV-sh-NC) or sh-METTL16 (LV-sh-METTL16, Genepharma, China) were infected in MDA-MB-231 cells and the infected cells were screened in DMEM with puromycin (3 µg/mL, Sigma-Aldrich) for 14 d to construct the stably cell of METTL16 knockdown. To build the murine subcutaneous tumor model, the stably cell line (10^6^ cells) was injected into mice (*n* = 6) subcutaneously for 4 weeks and the tumor volume was assessed at intervals of 1 weeks until the 28th day, when the mice were euthanized and the tumor tissues were taken for further study. For constructing metastasis model, the stably cell line (2 × 10^6^ cells) was injected through the tail vein into mice (*n* = 6) for 56 d, when the mice were euthanized and the lung tissues were taken. The experiments were supported by Dalian University of Technology.

### Hematoxylin and eosin (HE) staining

The 4% paraformaldehyde was utilized to fix the lung tissues and the paraffin was applied to embed the lung tissues. A 6 μm slice was obtained from the embedded tissues. The slices were incubated in hematoxylin for 3 min, and then dyed in eosin for 45 s. The slices were imaged under an AX-70 fluorescent microscope after dewatering.

### Immunohistochemical (IHC) staining

The 4% paraformaldehyde was utilized to fix the tumor tissues and the paraffin was applied to embed the tumor tissues. A 4 μm slice was obtained from the embedded tissues. Firstly, the slice was placed in H_2_O_2_ (3%) for 20 min and closed with goat serum in PBS for 35 min. Then, the slice was mixed with antibodies of anti-METTL16 (ab313743, 1:100, Abcam), anti-FBXO5 (ab187144, 1:100, Abcam), or anti-Ki67 (ab15580, 1:100, Abcam) at 4 °C for 12 h. The slice was cleaned in the PBS and then placed in the secondary antibody (ab150077, 1:500, Abcam) at 25 °C for 45 min. Finally, the slice was stained with DAB and hematoxylin and visualized under an AX-70 fluorescent microscope.

### Statistical analysis

The SPSS 26.0 software (SPSS, Inc.) was applied to treat the data, which was expressed as mean ± standard deviation (SD). The groups’ comparison and multiple groups’ comparison was analyzed by *t*-test and one-way ANOVA following Tukey’s test, respectively. All test were performed in triplicates at least. When the value of *P* < 0.05, the difference was identified as significance.

## Results

### METTL16 was overexpressed in BC

**Fig. 1:**
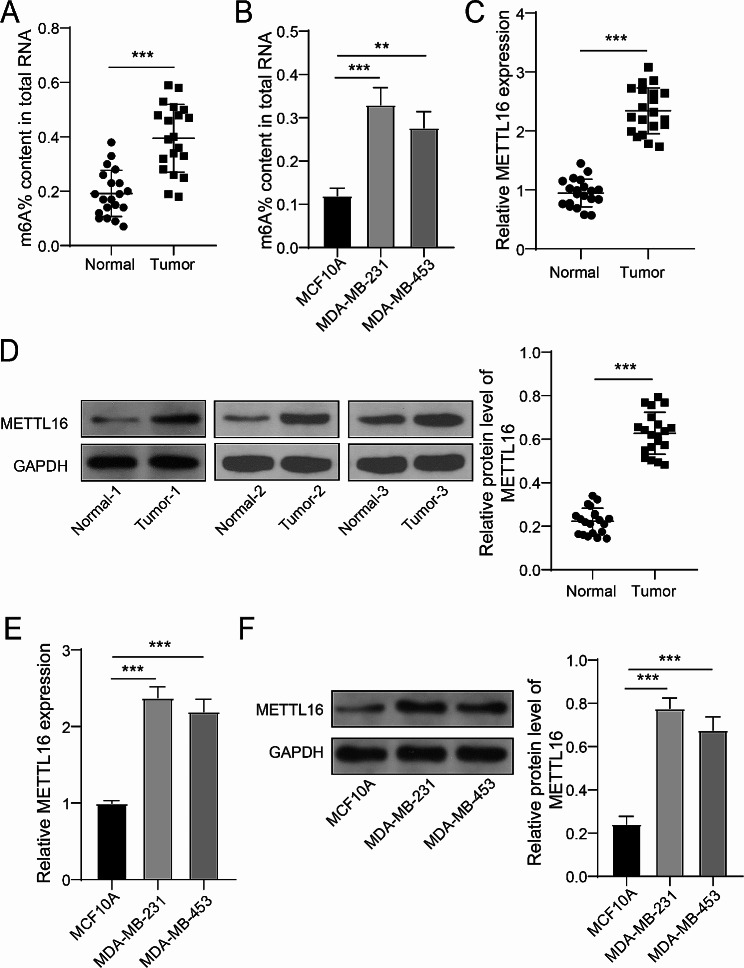
The expression of METTL16 was up-regulated in BC tissues and cells. (**A**, **B**) The overall m6A level of BC tissues and cells was tested using m6A RNA Methylation Assay kit. (**C**-**F**) The mRNA and protein levels of METTL16 in BC tissues and cells were tested using RT-qPCR and immunoblotting. **P < 0.01, ***P < 0.001

Firstly, the overall m6A level in BC tissues and cells (MDA-MB-231 and MDA-MB-453) was tested via m6A RNA Methylation Assay kit. As revealed in Fig. 1A and B, the overall m6A level of BC tissues and cells was higher than the normal tissues and breast epithelial cells (MCF10A). Further, the METTL16’s mRNA and protein levels in BC tissues were enhanced (Fig. 1C and D). Meanwhile, compared with MCF10A, the mRNA and protein expression of METTL16 in BC cells was also augmented (Fig. 1E and F). In conclusion, the levels of overall m6A and METTL16 were aggrandized in BC.

### METTL16 enhanced the malignant behavior of BC cells

**Fig. 2:**
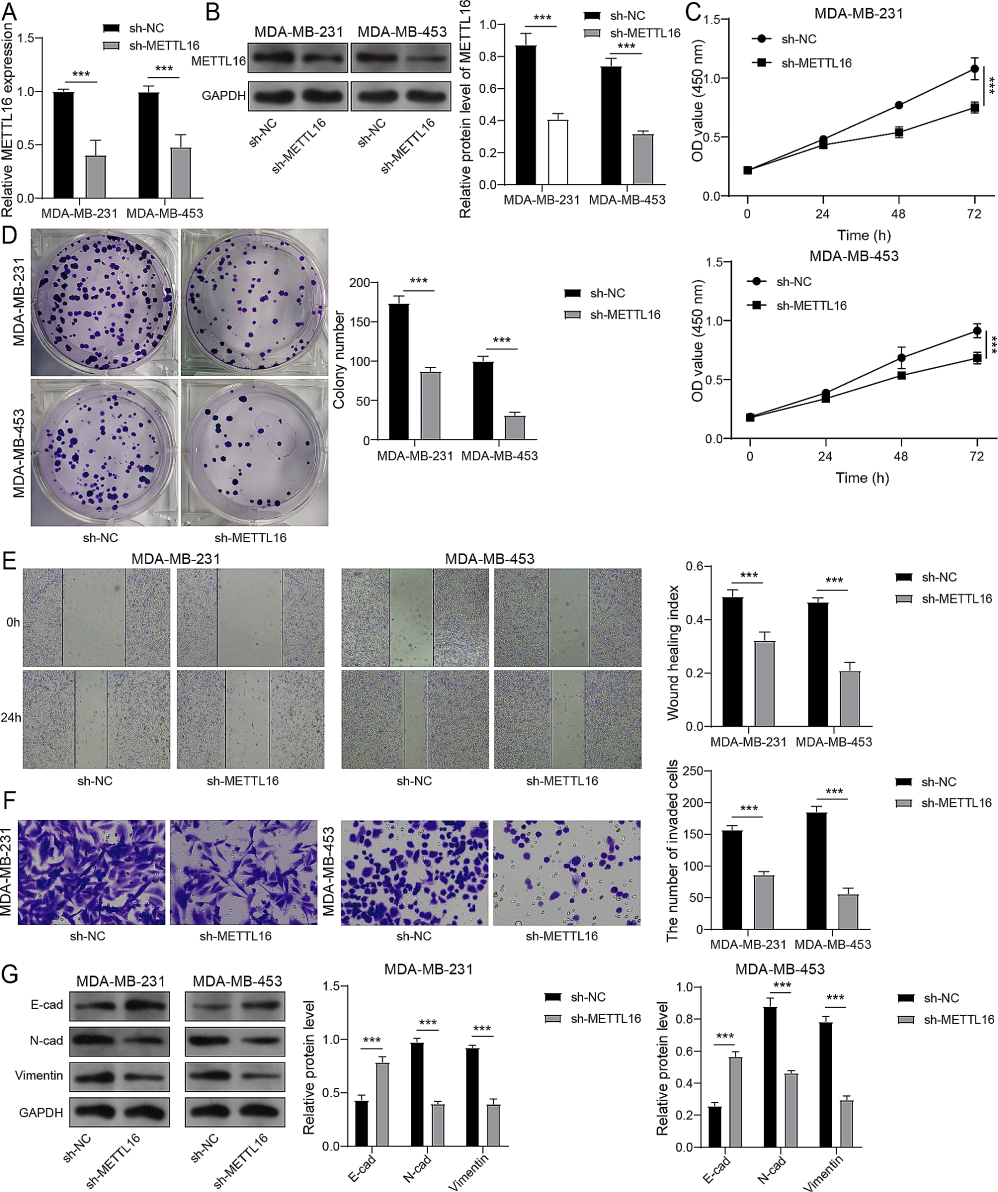
METTL16 enhanced the malignant behavior of BC cells. Knocking down METTL16 in MDA-MB-231 and MDA-MB-453 cells. (**A**, **B**) The mRNA and protein expression of METTL16 were monitored by RT-qPCR and immunoblotting. (**C**-**F**) The cell viability (**C**), colony forming ability (**D**), migration (**E**), and invasion (**F**) were tested by CCK-8 assay, clone formation assay, wound healing assay, and Transwell assay, respectively. (**G**) Immunoblotting was used to assess the expression of E-cad, N-cad, and Vimentin. ***P < 0.001

Then, we knocked down the METTL16 to explore the action of METTL16 in the progress of BC. The results of Fig. 2A and B indicated that the sh-METTL16 transfection lessened the level of METTL16, which manifested that METTL16 was successfully knocked down. METTL16 knockdown weakened the cell vitality and colony forming ability of BC cells (Fig. 2C and D). Meanwhile, the migration and invasion abilities of BC cells also restrained by knockdown of METTL16 (Fig. 2E and F). Furthermore, METTL16 knockdown aggrandized the level of E-cad and limited the expression of N-cad and Vimentin in BC cells (Fig. 2G), which revealed that the progress of epithelial-mesenchymal transition (EMT) was restricted by METTL16 knockdown. All in all, METTL16 knockdown restrained the BC cell’s proliferation, migration, invasion, and EMT.

### METTL16 accelerated the expression of FBXO5 in BC

**Fig. 3:**
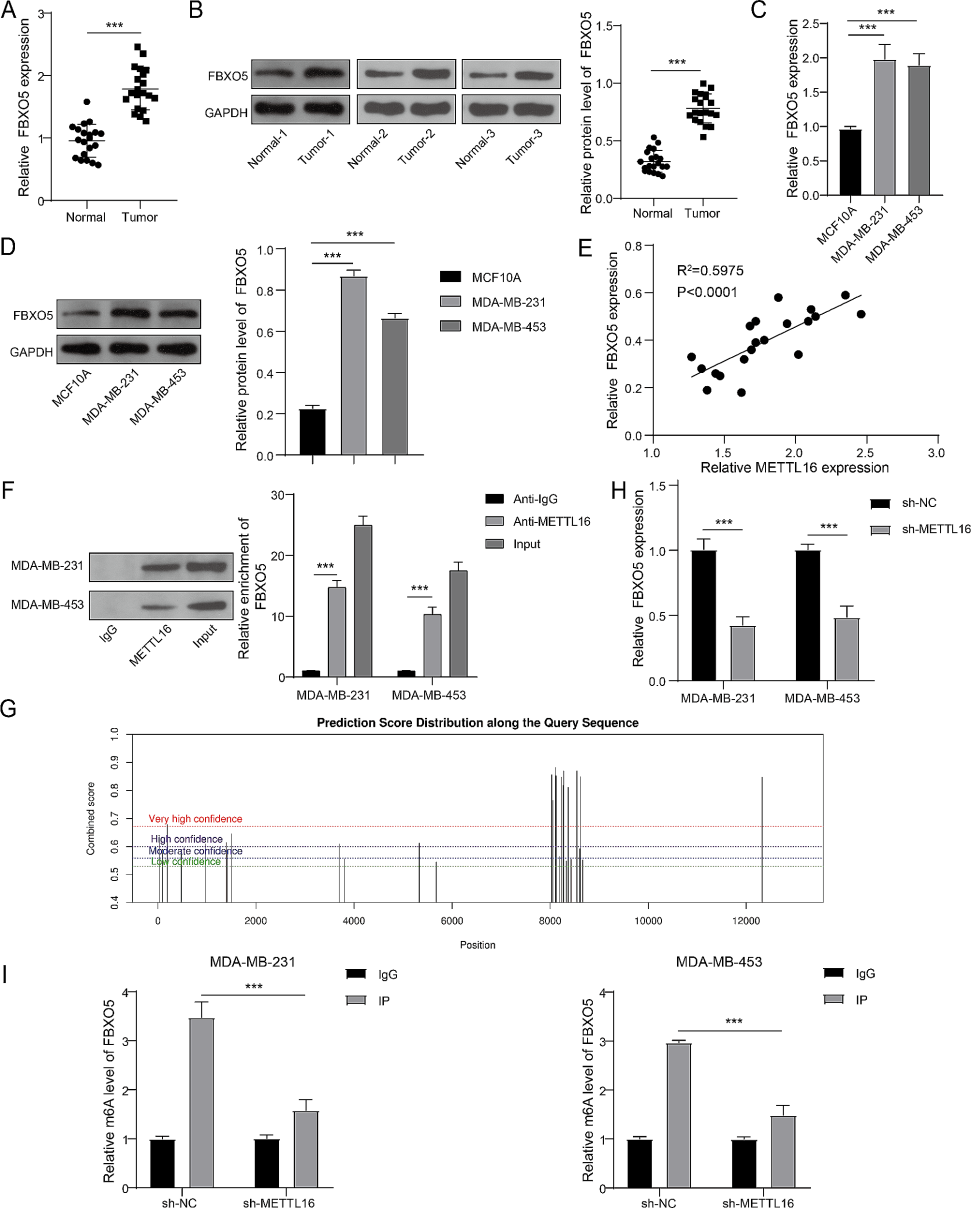
METTL16 accelerated the expression of FBXO5 in BC. (**A**-**D**) RT-qPCR and immunoblotting were utilized to estimate the mRNA and protein levels of FBXO5 in BC tissues and cells. (**E**) The expressed correlation between METTL16 and FBXO5 was assessed using Pearson correlation analysis. (**F**) RIP assay was performed to monitor the binding relationship between METTL16 and FBXO5 mRNA. (**G**) The potential m6A modification sites on FBXO5 mRNA was predicted using SRAMP database. (**H**) The mRNA level of FBXO5 in BC cells that were knockdown of METTL16 was tested using RT-qPCR. (**I**) MeRIP-qPCR assay was performed to assess the m6A level of FBXO5 in BC cells that were knockdown of METTL16. ***P < 0.001

The report showed that FBXO5 was upregulated in BC and relevant to poor prognosis [12, 18]. Here, we found that the FBXO5’s mRNA and protein levels in BC tissues and cells (MDA-MB-231 and MDA-MB-453) were higher than that in normal tissues and cells (Fig. 3A and D). Interestingly, the FBXO5 level and METTL16 level was positively correlated (Fig. 3E). Meanwhile, the MAT2A was selected as positive control of RIP [19] and the GAPDH was selected as negative control of RIP. The results showed that the antibody of METTL16 enriched the FBXO5 mRNA (Fig. 3F) and MAT2A mRNA (Supplementary Fig. 1) in BC cells, indicated the binding relationship between METTL16 and FBXO5 mRNA. Otherwise, the SRAMP database forecasted the latent m6A sites on FBXO5 mRNA (Fig. 3G). Further, knockdown of METTL16 restrained the level of FBXO5 mRNA and the m6A level of FBXO5 mRNA in BC cells (Fig. 3H and I). Overall, the expression of FBXO5 was positively regulated by METTL16 in BC.

### METTL16 facilitated the mRNA stability of FBXO5 in BC

**Fig. 4:**
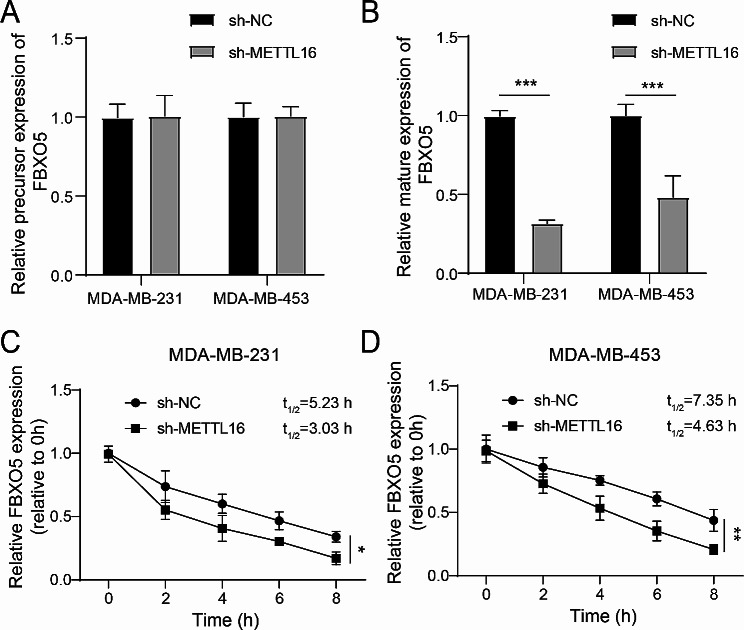
METTL16 facilitated the mRNA stability of FBXO5 in BC. Knocking down METTL16 in MDA-MB-231 and MDA-MB-453 cells. (**A**, **B**) RT-qPCR was applied to test the precursor and mature level of FBXO5 mRNA. The MDA-MB-231 and MDA-MB-453 cells were treated with actinomycin D (2 µg/mL) for 0, 2, 4, 6, 8 h, respectively. (C, D) The mRNA level of FBXO5 was assessed using RT-qPCR. *P < 0.05, **P < 0.01, ***P < 0.001

Subsequently, we knocked down METTL16 in BC cells and tested the FBXO5 mRNA’s precursor (FBXO5 pre-mRNA) and the mature FBXO5 mRNA level. The data indicated that METTL16 knockdown weakened the mature FBXO5 mRNA level but did not affect the level of FBXO5 pre-mRNA (Fig. 4A and B). Furthermore, to assess the impact of METTL16 on the FBXO5 mRNA’s stability, after knockdown of METTL16, the BC cells were treated with actinomycin D for 0, 2, 4, 6, 8 h, respectively. As revealed in Fig. 4C and D, METTL16 knockdown accelerated the decrease of FBXO5 mRNA. Thus, the mRNA stability of FBXO5 in BC was strengthened by METTL16.

### METTL16 boosted the BC cell’s malignant action via regulating FBXO5

**Fig. 5:**
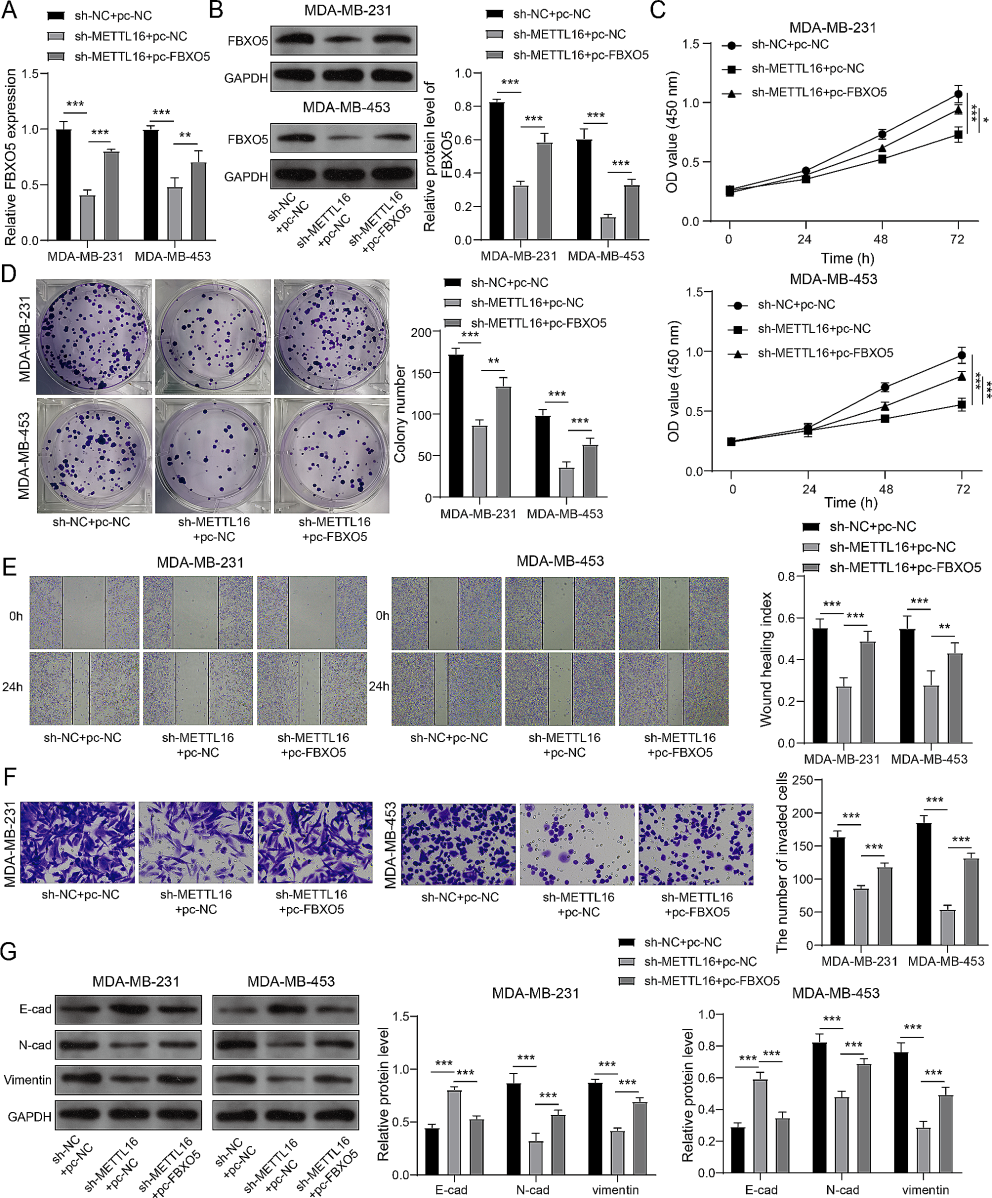
METTL16 boosted the malignant behavior of BC cells via regulating FBXO5. The MDA-MB-231 and MDA-MB-453 cells were knocked down METTL16 and overexpressed FBXO5. (**A**, **B**) RT-qPCR and immunoblotting was applied to measure the mRNA and protein expression of FBXO5. (**C**-**F**) The cell viability (**C**), colony forming ability (**D**), migration (**E**), and invasion (**F**) were tested by CCK-8 assay, clone formation assay, wound healing assay, and Transwell assay, respectively. (**G**) Immunoblotting was used to assess the expression of E-cad, N-cad, and Vimentin. *P < 0.05, **P < 0.01, ***P < 0.001

To assess whether the METTL16 mediates the BC cell’s malignant action via regulating FBXO5, we knocked down METTL16 and/or overexpressed FBXO5 (Supplementary Fig. 2) in BC cells. The data of Fig. 5A and B revealed that overexpression of FBXO5 restrained the decrease in mRNA and protein expression of FBXO5 caused by sh-METTL16 transfection, which demonstrated that FBXO5 was overexpressed successfully. METTL16 knockdown weakened the BC cell’s vitality and colony forming ability, while FBXO5 overexpression reversed this effect (Fig. 5C and D). Furthermore, overexpression of FBXO5 also augmented the BC cell’s migration and invasion abilities that were suppressed by METTL16 knockdown (Fig. 5E and F). Meanwhile, knockdown of METTL16 accelerated the E-cad’s expression and lessened the expression of N-cad and Vimentin in MDA-MB-231 and MDA-MB-453 cells, while overexpression of FBXO5 counteracted this impact (Fig. 5G), which indicated that the EMT progress was restricted by METTL16 knockdown and facilitated by FBXO5 overexpression. In general, METTL16 motivated the BC cell’s proliferation, migration, invasion, and EMT by increasing FBXO5.

### METTL16 accelerated the growth and metastasis of BC in vivo

**Fig. 6:**
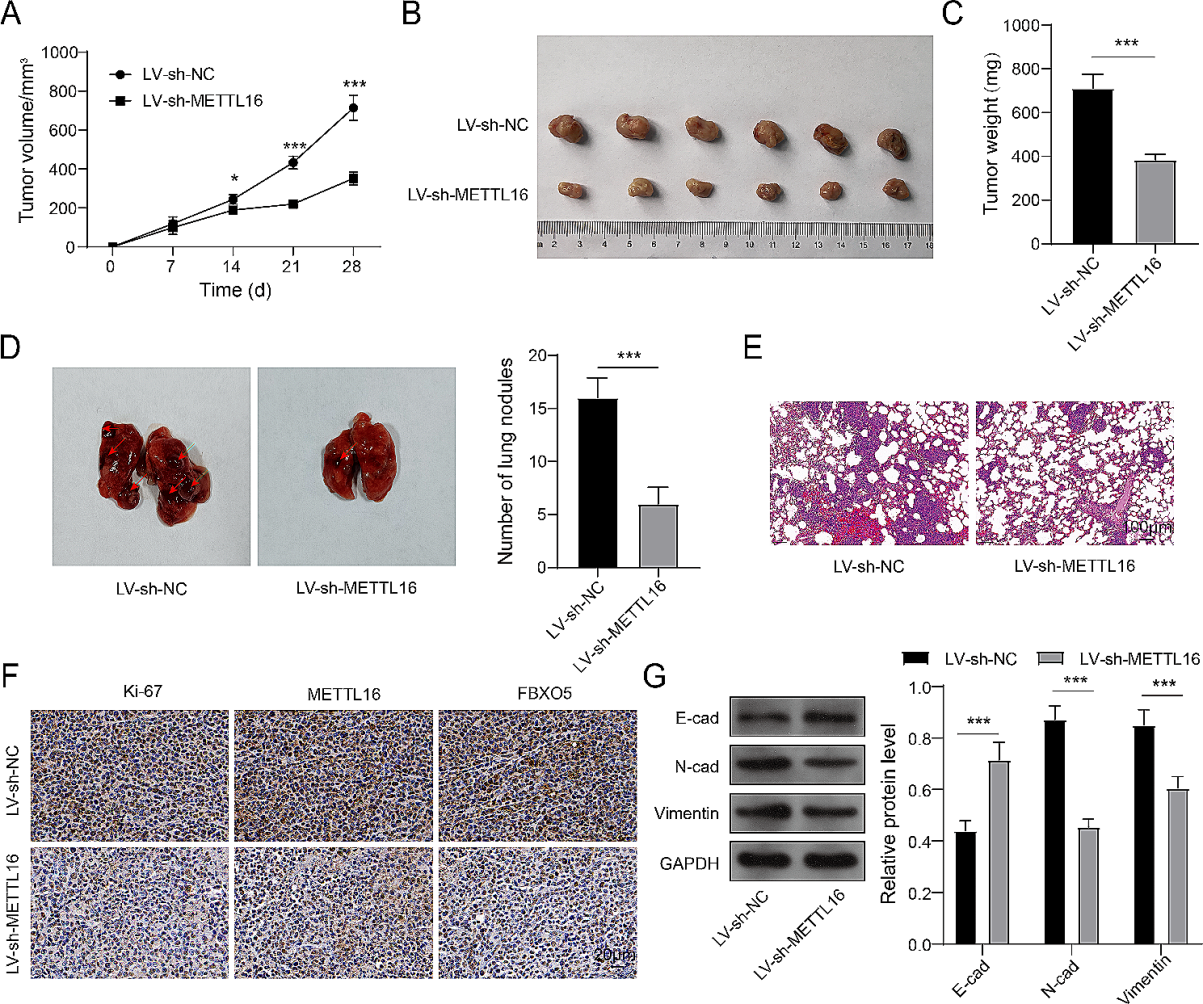
METTL16 accelerated the growth and metastasis of BC in vivo. The murine subcutaneous tumor model and the metastasis model were constructed by injecting the stably cell line of METTL16 knockdown subcutaneously or via the tail vein. (**A**) The volume of tumor was assessed. (**B**) Representative images of tumors. (**C**) The weight of tumor was tested. (**D**) The number of pulmonary metastatic nodules were observed. (**E**) HE staining was employed to detect the lung metastasis. (**F**) The expression of Ki-67, METTL16, and FBXO5 was monitored using IHC. (**G**) Immunoblotting was performed to test the expression of E-cad, N-cad, and Vimentin. *P < 0.05, ***P < 0.001

To further expound the cancer-promoting function of METTL16, the murine subcutaneous tumor and the metastasis model were built via injecting the stably cell line of METTL16 knockdown subcutaneously or via the tail vein. The Fig. 6A and C indicated that the volume and weight of tumors in the METTL16 knockdown group were obviously lower than those in the control group. Simultaneously, the number of lung metastatic nodules decreased in the group of METTL16 knockdown (Fig. 6D and E). Moreover, METTL16 knockdown lessened the number of cell that Ki-67 positive, METTL16, and FBXO5 (Fig. 6F). Otherwise, enhanced E-cad’s expression and reduced expression of N-cad and Vimentin were present in tumor tissues of the METTL16 knockdown group (Fig. 6G). Overall, the growth and metastasis of BC were restrained by knockdown of METTL16.

## Discussion

BC is the most common malignancy in the world and the leading cause of cancer-related death in women [20, 21]. Although advances in current treatment regimens have greatly improved the prognosis of BC patients, the occurrence of metastasis in BC is still a major obstacle to effective treatment [2, 22]. The cell invasion and migration perform a crucial function in the progress of metastasis in BC [23]. Here, we found that METTL16 knockdown can decrease the proliferation, migration, invasion, and EMT of BC cells via regulating the FBXO5’s expression, and thereby restrain the growth and metastasis of BC in vivo, suggesting their potential as therapeutic targets for inhibiting BC progression and metastasis.

The m6A modification of RNA has been widely studied for its promoting effects on proliferation, migration, invasion, and EMT in cancer cells, and even the growth and metastasis of tumors in vivo in various cancer [24–26]. There is evidence suggesting that inhibition of the m6A modification of RNA can limit the proliferation, migration, invasion, and in vivo tumor metastasis of BC [24, 27, 28]. In our research, we discovered an overall enhancement in m6A modification levels of total RNA in BC tissues and cells, which is consistent with previous research findings [29]. Furthermore, we observed that METTL16, a key regulator of m6A modification, was upregulated in BC cells and tissues, which aligns with the findings of Ye et al. [11], who proved METTL16 was upregulated in tumor tissues from BC patients and facilitated BC progression by restraining ferroptosis via regulating GPX4. Not exactly as has been reported [11], we discovered that inhibition of METTL16 suppressed BC progress via lessening the proliferation, migration, invasion, and EMT processes of BC cells, inhibiting the growth and metastasis of tumors in vivo, which is reported for the first time.

FBXO5 is an important cell cycle regulation factor [30, 31]. Nevertheless, FBXO5’s function in BC remains unclear. Our research demonstrated that FBXO5 is overexpressed in BC tissues, which consistent with previous reports [17, 18, 32]. Additionally, we have made the novel discovery of a positive correlation between the expression of FBXO5 and METTL16 in BC tissues. METTL16 interacted with FBXO5 mRNA, increasing the mRNA stability of FBXO5 depending on m6A. The research have shown that FBXO5, which is regulated by transcription factor ELF1, enhances the proliferation, migration, and invasion of gliomas [16]. Our research found that inhibition of METTL16 weakens the proliferation, migration, invasion, and EMT process of BC cells, while FBXO5 overexpression reverses these effects, suggesting the oncogenic role of FBXO5 in BC.

## Conclusions

In general, our findings discovered that METTL16 enhanced the mRNA stability of FBXO5 via m6A modification to accelerate the proliferation, migration, invasion, and EMT of BC cells, and thereby facilitate the growth and metastasis of BC in vivo. Our present research offers new latent targets for the diagnosis and cure of BC.

### Electronic supplementary material

Below is the link to the electronic supplementary material.

Supplementary Figure 1: The binding relationship between METTL16 and MAT2A/GAPDH/FBXO5 (A, B) RIP-qPCR assay was performed to assess the binding relationship between METTL16 and MAT2A/GAPDH/FBXO5 in BC cells. ***P 0.001

Supplementary Figure 2: Determination of FBXO5 transfection efficiency. The MDA-MB-231 and MDA-MB-453 cells were overexpressed FBXO5. (A, B) RT-qPCR and immunoblotting was applied to measure the mRNA and protein expression of FBXO5 in BC cells. ***P 0.001

## Data Availability

The datasets used or analyzed during this study can be made available from the corresponding author upon reasonable request.
