# Supplementary figures and images for: METTL16 regulates the mRNA stability of FBXO5 via m6A modification to facilitate the malignant behavior of breast cancer
Source: Cancer Metab. 2024 Jul 25;12:22. doi: 10.1186/s40170-024-00351-5 (PMC11282785; doi:10.1186/s40170-024-00351-5)

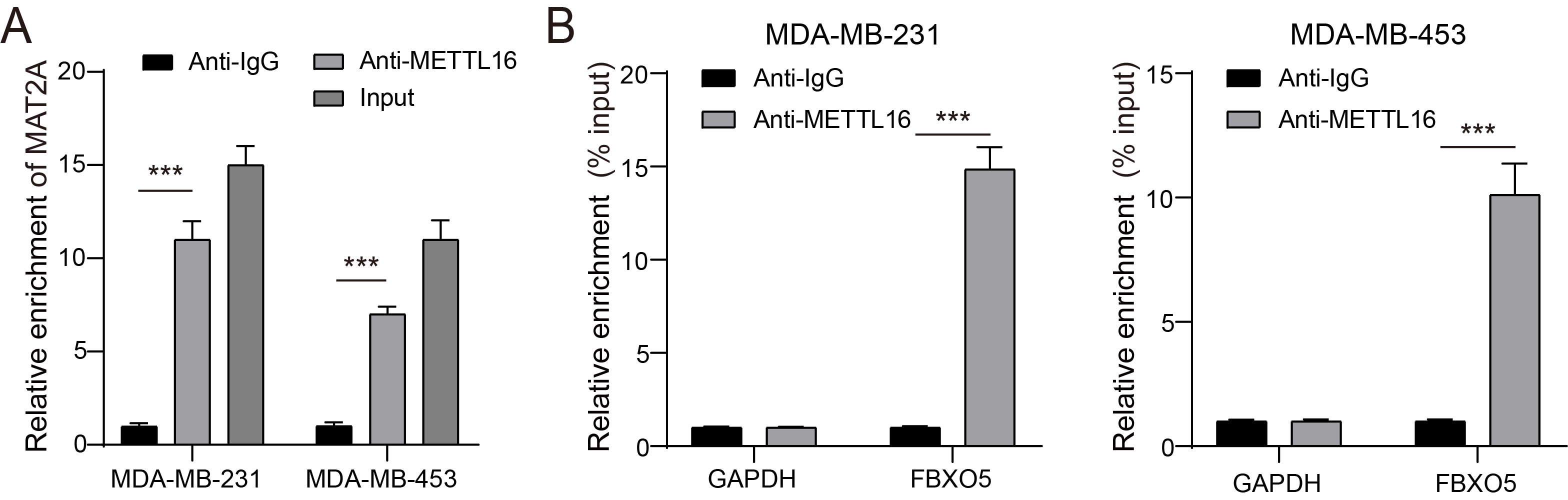

Supplement: Supplementary file 1 — Supplementary Figure 1: The binding relationship between METTL16 and MAT2A/GAPDH/FBXO5 (A, B) RIP-qPCR assay was performed to assess the binding relationship between METTL16 and MAT2A/GAPDH/FBXO5 in BC cells. ***P 0.001 [file 40170_2024_351_MOESM1_ESM.tif]

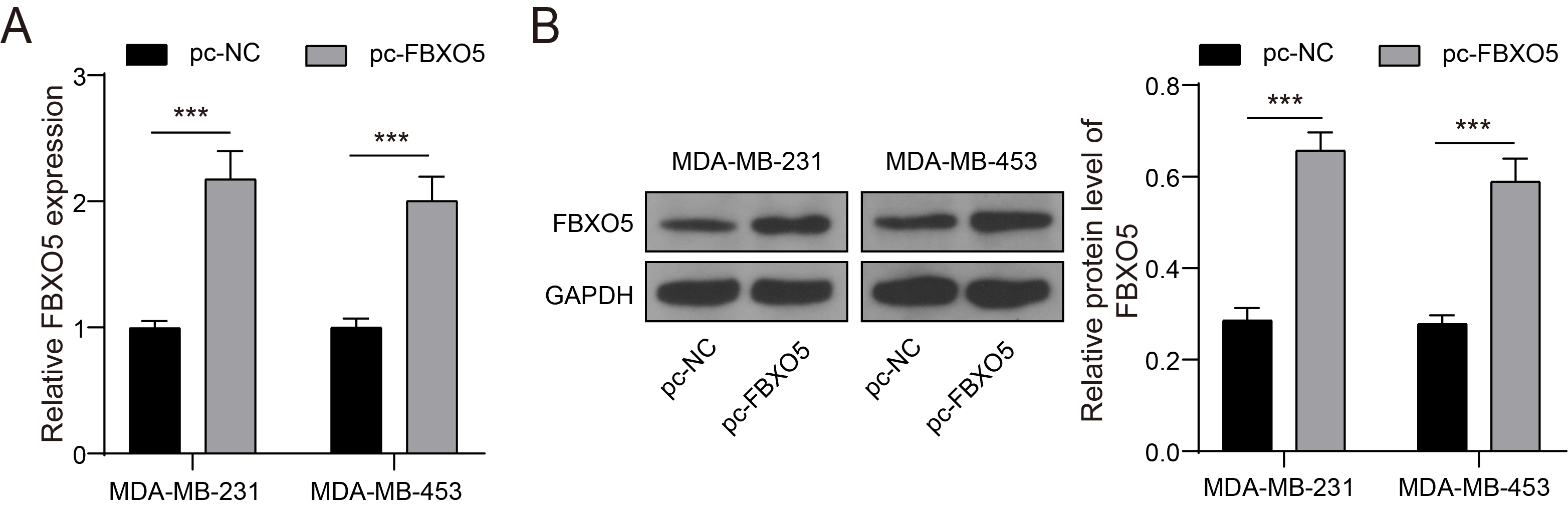

Supplement: Supplementary file 2 — Supplementary Figure 2: Determination of FBXO5 transfection efficiency. The MDA-MB-231 and MDA-MB-453 cells were overexpressed FBXO5. (A, B) RT-qPCR and immunoblotting was applied to measure the mRNA and protein expression of FBXO5 in BC cells. ***P 0.001 [file 40170_2024_351_MOESM2_ESM.tif]
